# Supplementary material for: Heat adaptation of phage T7 under an extended genetic code
Source: Virus Evol. 2021 Dec 1;7(2):veab100. doi: 10.1093/ve/veab100 (PMC8923235; doi:10.1093/ve/veab100)
Supplement: veab100_Supp [file veab100_supp.zip › Sequencing_Analysis.zip!/Sequencing_Analysis/A3/evidence/SNP_12.html]

BRESEQ :: Evidence


| Predicted mutation | | | | | | |
| --- | --- | --- | --- | --- | --- | --- |
| evidence | seq id | position | mutation | annotation | gene | description |
| RA | NC\_001604.1 | 28,692 | A→G | intergenic (–/–) | – / – | –/– |

| Read alignment evidence... | | | | | | | | | | | |
| --- | --- | --- | --- | --- | --- | --- | --- | --- | --- | --- | --- |
|  | seq id | position | | ref | new | freq | score (cons/poly) | reads | annotation | genes | product |
| \* | NC\_001604.1 | 28,692 | 0 | A | G | 99.1% | 4773.2 / ‑4.7 | 1586 | intergenic (–/–) | *–/–* | –/– |
| Reads supporting (aligned to +/- strand):  **ref** base A (1/2);  **major** base G (793/776);  **minor** base T (8/6);  **total** (802/784) | | | | | | | | | | | |
| Fisher's exact test for biased strand distribution *p*-value = 7.90e-01 | | | | | | | | | | | |
| Kolmogorov-Smirnov test that lower quality scores support variant *p*-value = 9.39e-01 | | | | | | | | | | | |

Only 100 of 1603 total aligned reads displayed.  

`GGTATGGGCTATCGTGCAGCAACCACTCAGGCCGAACAGCCAAGGTCAAGCCTATTGGACACCATTGGTCGGTTCGCTAAGGCTGGTGCCGATATGTATACCGCTAAGGAACAACGAGCACGAGACCTAGCTGATGAACGCTCTAACGAGATTATCCGTAAGCTGACCCCTGAGCAACGTCGAGAAGCTCTCAACAACGGGACCCTTCTGTATCAGGATGACCCATACGCTATGGAAGCACTCCGAGTCAAGACTGGTCGTAACGCTGCGTATCTTGTGGACGATGACGTTATGCAGAAGATAAAAGAGGGTGTCTTCCGTACTCGCGAAGAGATGGAAGAGTATCGCCATAGTCGCCTTCAAGAGGGCGCTAAGGTATACGCTGAGCAGTTCGGCATCGACCCTGAGGACGTTGATTATCAGCGTGGTTTCAACGGGGACATTACCGAGCGTAACATCTCGCTGTATGGTGCGCATGATAACTTCTTGAGCCAGCAAGCTCAGAAGGGCGCTATCATGAACAGCCGAGTGGAACTCAACGGTGTCCTTCAAGACCCTGATATGCTGCGTCGTCCAGACTCTGCTGACTTCTTTG  >  NC_001604.1/28394‑28988`  

                                                                                                                                                                                                                                                                                                          `|`                                                                                                                                                                                                                                                                                                          
      `ggCTATCGTGCAGCAACCACTCAGGCCGAACAGCCAAGGTCAAGCCTATTGGACACCATTGTTCGGTTCGCCAAGGCTGGTGCCGATATGTATACCGCTAAGGAACAACGAGCACGAGACCTAGCTGATGAACGCTCTAACGAGATTATCCGTAAGCTGACCCCTGAGCAACGGCGAGAAGCTCTCAACAACGGGACCCTTCTGTATCAGGATGACCCATACGCTATGGAAGCACTCCGAGTCAAGACTGGTCGTAACGCTGCGTATCTTGTGGACGATGACGTTATGCAGAGGATAAAag                                                                                                                                                                                                                                                                                                  <  1:71529/301‑1 (MQ=255)`  
      `ggCGATCGTGCAGCAACCACTCAGGCCGAACAGCCAAGGTCAAGCCTATTGGACACCATTGGTCGGTTCGCTAAGGCTGGTGCCGATATGTATACCGCTAAGGAACAACGAGCACGAGACCTAGCTGATGAACGCTCTAACGAGATTATCCGTAAGCTGACCCCTGAGCAACGTCGAGAAGCTCTCAACAACGGGACCCTTCTGTATCAGGATGACCCATACGCTATGGAAGCACTCCGAGTCAAGACTGGTCGTAACGCTGCGTATCTTGTGGACGATGACGTTATGCAGAGGATAAAag                                                                                                                                                                                                                                                                                                  <  1:10560/301‑1 (MQ=255)`  
      `gagacaggTGCAGCAACCACTCAGGCCGAACAGCCAAGGTCAAGCCTATTGGACACCATTGGTCGGTTCGCTAAGGCTGGTGCCGATATGTATACCGCTAAGGAACAACGAGCACGAGACCTAGCTGATGAACGCTCTAACGAGATTATCCGTAAGCTGACCCCTGAGCAACGTCGAGAAGCTCTCAACAACGGGACCCTTCTGTATCAGGATGACCCATACGCTATGGAAGCACTCCGAGTCAAGACTGGTCGTAACGCTGCGTATCTTGTGGACGATGACGTTATGCAGAGGATAAAag                                                                                                                                                                                                                                                                                                  <  1:24029/294‑1 (MQ=255)`  
          `aTCGTGCAGCAACCACTCAGGCCGAACAGCCAAGGTCAAGCCTATTGGACACCATTGGTCGGTTCGCTAAGGCTGGTGCCGATATGTATACCGCTAAGGAACAACGAGCACGAGACCTAGCTGATGAACGCTCTAACGAGATTATCCGTAAGCTGACCCCTGAGCAACGTCGAGAAGCTCTCAACAACGGGACCCTTCTGTATCAGGATGACCCATACGCTATGGAAGCACTCCGAGTCAAGCCTGGTCGTAACGCTGCGTATCTTGTGGACGATGACGTTATGCAGAGGATAAAagctgt                                                                                                                                                                                                                                                                                              >  1:108686/1‑297 (MQ=255)`  
            `cGTGCAGCAACCACTCAGGCCGAACAGCCAAGGTCAAGCCTATTGGACACCATTGGTCGGTTCGCTAAGGCTGGTGCCGATATGTATACCGCTAAGGAACAACGAGCACGAGACCTAGCTGATGAACGCTCTAACGAGATTATCCGTAAGCTGACCCCTGAGCAACGTCGAGAAGCTCTCAACAACGGGACCCTTCTGTATCAGGATGACCCATACGCTATGGAAGCACTCCGAGTCAAGACTGGTCGTAACGCTGCGTATCTTGTGGACGATGACGTTATGCAGAGGATAAAAGAGGgtg                                                                                                                                                                                                                                                                                            <  1:304091/301‑1 (MQ=255)`  
                         `cTCAGGCCGAACAGCCAAGGTCAAGCCTATTGGACACCATTGGTCGGTTCGCTAAGGCTGGTGCCGATATATATACCGCTAAGGAACAACGAGCACGAGACCTAGCTGATGAACGCTCTAACGAGATTATCCGTAAGCTGACCCCTGAGCAACGTCGAGAAGCTCTCAACAACGGGACCCTTCTGTATCAGGATGACCCATACGCTATGGAAGCACTCCGAGTCAAGACTGGTCGTAACGCTGCGTATCTTGTGGACGATGACGTTATGCAGAGGATAAAAGAGGGTGTCTTCCGTACTcg                                                                                                                                                                                                                                                                               <  1:100136/301‑1 (MQ=255)`  
                             `ggCCGAACAGCCAAGGTCAAGCCTATTGGACACCATTGGTCGGTTCGCTAAGGCTGGTGCCGATATGTATACCGCTAAGGAACAACGAGCACGAGACCTAGCTGATGAACGCTCTAACGAGATTATCCGTAAGCTGACCCCTGAGCAACGTCGAGAAGCTCTCAACAACGGGACCCTTCTGTATCAGGATGACCCATACGCTATGGAAGCACTCCGAGTCAAGACTGGTCGTAACGCTGCGTATCTTGTGGACGATGACGTTATGCAGAGGATAAAAGAGGGTGTCTTCCTTACTCGCGaa                                                                                                                                                                                                                                                                           >  1:37463/1‑301 (MQ=255)`  
                                      `gCCAAGGTCAAGCCTATTGGACACCATTGGTCGGTTCGCTAAGGCTGGTGCCGATATGTATACCGCTAAGGAACAACGAGCACGAGACCTAGCTGATGAACGCTCTAACGAGATTATCCGTAAGCTGACCCCTGAGCAACGTCGAGAAGCTCTCAACAACGGGACCCTTCTGTATCAGGATGACCCATACGCTATTGAAGCACTCCGAGTCAAGACTGGTCGTAACGCTGCGTATCTTGTGGACGATGACGTTATGCAGAGGATAAAAGAGGGTGTCTTCCGTAcctgtctcttatacaca                                                                                                                                                                                                                                                                  >  1:252752/1‑285 (MQ=255)`  
                                      `gCCAAGGTCAAGCCTATTGGACACCATTGGTCGGTTCGCTAAGGCTGGTGCCGATATGTATACCGCTAAGGAACAACGAGCACGAGACATAGCTGATGAACGCTCTAACGAGATTATCCGTAAGCTGACCCCTGAGCAACGTCGAGAAGCTCTCAACAACGGGACCCTTCTGTATCAGGATGACCCATACGCTATGGAAGCACTCCGAGTCAAGACTGGTCGT‑ACGCTGCGTATCTTGTGGACGATGACGTTATGCAGAGGATAAAAGAGGGTGTCTTcctgtctcttatacacatctccg                                                                                                                                                                                                                                                                 >  1:254445/1‑280 (MQ=255)`  
                                        `cAAAGTCAAGCCTATTGGACACCATTGGTAGGTTCGCTAAGGCTGGTGCCGATATGTATACCGCTAAGGAACAACGAGCACGAGACCTAGCTGATGAACGCTCTAACGAGATTATCCGTAAGCTGACCCCTGAGCAACGTCGAGAAGCTCTCAACAACGGGACCCTTCTGTATCAGGATGACCAATACGCTATGGAAGCACTCCGAGTCAAGACTGGTCGTAACGCTGCGTATCTTGTGGACGATGACGTTATGCAGAGGATAAAAGAGGGTGTCTTCCGTACTCGCGAAGAGATGGAaga                                                                                                                                                                                                                                                                <  1:298879/301‑1 (MQ=255)`  
                                           `ggTCAAGCCTATTGGACACCATTGGTCGGTTCGCTAAGGCTGGTGCCGATATGTATACCGCTAAGGAACAACGAGCACGAGACCTAGCTGATGAACGCCCTAACGAGATTATCCGTAAGCTGACCCCTGAGCAACGTCTAGAAGCTCTCAACAACGGGACCCTTCTGTACCAGGATGACCCATACGCTATGGAAGCACTCCGAGTCAATACTGGTCGTAACGCTGCGTATCGTGTGGATGATGACGTTATGCAGAGGATAAAAGAGGGTGTCTTCCGTACTCGGGAAGAGATGGAagttgt                                                                                                                                                                                                                                                             >  1:102590/1‑297 (MQ=255)`  
                                            `acagcaccTATTGGACACCATTGGTCGGTTAGCTAAGGCTGGTGCCGATATGTATACCGCTAAGGAACAACGAGCACGAGACCTAGCTGATGAACGCTCTAACGAGATTATCCGTAAGCTGACCCCTGAGCAACGTCGAGAAGCTCTCAACAACGGGACCCTTCTGTATCAGGATGACCCATACGCTATGGAAGCACTCCGAGTCAAGACTGGTCGTAACGCTGCGTATCTTGTGGACGATGACGTTATGCAGAGGATAAAAGAGGGTGTCTTCCGTACTCGCGAAGAGATGGAAGAGTAt                                                                                                                                                                                                                                                            <  1:274813/295‑1 (MQ=255)`  
                                                 `gCCTATTGTACACCATTGGTCGGTTCGCTAAGGCTGGTGCCGATATGTATACCGCTAAGGAACAACGAGCACGAGACCTAGCTGATGAACGCTCTAACGAGATTATCCGTAAGCTGACCCCTGAGCAACGTCGAGAAGCTCTCAACAACGGGACCCTTCTGTATCAGGATGACCCATACGCTATGGAAGCACTCCGAGTCAAGACTGGTCGTAACGCTGCGTATCTTGTGGACGATGACGTTATGCAGAGGATAAACGAGGGTGTCTTCCGTACTCGCGAAGAGATGGAAGAGTATCGccc                                                                                                                                                                                                                                                       >  1:319329/1‑300 (MQ=255)`  
                                                  `aagaTTGGACACCATTGGTCGGTTCGCTAAGGCTGGTGCCGATATGTATACCGCTAAGGAACAACTAGAACGATACCTAGCTGATTAACGCTCTAACGAGATTATCCGTAAGCTGACCCCTGAGCAACGTCGAGAAGCTCTCAACAACGGGACCATTCTGTATCAGGATGACCCATACGCTATGGAAGCACTTCGAGTCAAGACTGGTCGTAACGCTGCGTATCTTGTGGACGATGACGTTATGCAGAGGATAAAAGAGGGTGTCTTCCGTACTCGCGAAGAGATGGAAGAGTATCGCCAt                                                                                                                                                                                                                                                      <  1:24333/298‑1 (MQ=255)`  
                                                   `cTATTGGACACCATTGGTCGGTTCGCTAAGGCTGGTGCCGATATGTATACCGCTAAGGAACAACGAGCACGAGACCTAGCTGATGAACGCTCTAACGAGATTATCCGTAAGCTGACCCCTGAGCAACGTCGAGAAGCTCTCAACAACGGGACCCTTCTGTATCAGGATGACCCATACGCTATGGAAGCACTCCGAGTCAAGACTGGTCGTAACGCTGCGTATCTTGTGGACGATGACGTTATGCAGAGGATAAAAGAGGGTGTCTTCCGTACTCGCTAAGAGATGGAAGAGTATCGCCATa                                                                                                                                                                                                                                                     >  1:131918/1‑301 (MQ=255)`  
                                                     `aTTGGACACCATTGGTCGGTTCGCTAAGGCTGGTGCCGATATGTATACCGCTAAGGAACAACGAGCACGAGACCTAGCTGATGAACGCTCTAACGAGATTATCCGTAAGCTGACCCCTGAGCAACGTCGAGAAGCTCTCAACAACGGGACCCTTCTGTATCAGGATGACCCATACGCTATGGAAGCACTCCGAGTCAAGACTGGTCGTAACGCTGCGTATCTTGTGGACGATGACGTTATGCAGAGGATAAAAGAGGGTGTCTTCCGTACTCGCGAAGAGCTggctcttagacacatctcc                                                                                                                                                                                                                                                   >  1:217763/1‑284 (MQ=255)`  
                                                        `ggACACCATTGGTCGGTTCGCTAAGGCTGGTGCCGATATGTATACCGCTAAGGAACAACGAGCACGAGACCTAGCTGATGAACGCTCTAACTAGATTATCCGTAAGCTGACCCCTGAGCAACGTCGAGAAGCTCTCAACAACGGGACCCTTCTGTATCAGGATGACCCATACGCTATGGAAGCACTCCGAGTCAAGACTGGTCGTAACGCTGCGTATCTTGTGGACGATGACGTTATGCAGAGGATAAAAGAGGGTGTCTTCCGTACTCGCGAAGAGATGGAAGAGTATCGCCATAGTCGc                                                                                                                                                                                                                                                >  1:170537/1‑301 (MQ=255)`  
                                                             `ccATTGGTCGGTTCGCTAAGGCTGGTGCCGATATGTATACCGCTAAGGAACACCGAGCACGAGACCTAGCTGATGACCGCTCTAACGAGATTATCCGTAAGCTGACCCCTGAGCAACGTCGAGAAGCTCTCAACAACGGGACCCTTCTGTATCAGGATGACCCATACGCTATGGAAGCACTCCGAGTCAAGACTGGTCGTACCGCTGCGTATCTTGTGGACGATGACGTTATGCCGAGGATAAAAGAGGGTGTCTTCCGTACTCGCGAAGAGATGGAAGAGTATctgcttcttatacacat                                                                                                                                                                                                                                           >  1:246194/1‑285 (MQ=255)`  
                                                             `ccATTGGTCGGTTCGCTAAGGCTGGTGCCGATATGTATACCGCTAAGGAACAACGAGCACGAGACCTAGCTGATGAACGCTCTAACGAGATTATCCGTAAGCTGACCCCTGAGCAACGTCGAGAAGCTCTCAACAACGGGACCCTTCTGTATCAGGATGACCCATACGCTATGGAAGCACTCCGAGTCAAGACTGGTCGTAACGCTGCGTATCTTGTTGACGATGACGTTATGCAGAGGATAAAAGATGGTGTCTTCCGTACTCGCGAAGAGATGGAagctgtctcttatacacatctccg                                                                                                                                                                                                                                           >  1:9712/1‑279 (MQ=255)`  
                                                             `ccATTGGTCGGTTCGCTAAGGCTGGTGCCGATATGTATACCGCTAAGGAACAACGAGCACGAGACCTAGCTGATGAACGCTCTAACGAGATTATCCGTAAGCTGACCCCTGAGCAACGTCGAGAAGCTCTCAACAACGGGACCCTTCTGTATCAGGATGACCCATACGCTATGGAAGCACTCCGAGTCAAGACTGGTCGTAACGCTGCGTATCTTGTGGACGATGACGTTATGCAGAGGATAAAAGAGGGTGTCTTCCGTACTCGCGAAGAGATGGAAGAGTATCGCCactgtctcttata                                                                                                                                                                                                                                           >  1:110921/1‑289 (MQ=255)`  
                                                                                `ggCTGGTGCCGATATGTATACCGCTAAGGAACAACGAGCACGAGACCTAGCTGATGAACGCTCTAACGAGATTATCCGTAAGCTGACCCCTGAGCAACGTCGAGAAGCTCTCAACAACGGGACCCTTCTGTATCAGGATGACCCATACGCTATGGAAGCACTCCGAGTCAAGACTGGTCGTAACGCTGCGTATCTTGTGGACGATGACGTTATGCAGAGGATAAAAGAGGGTGTCTTCCGTACTCGCGAAGAGATGGAAGAGTATCGCCATAGTCGctgtctcttatacacatctccgagc                                                                                                                                                                                                                        >  1:43651/1‑277 (MQ=255)`  
                                                                                  `cTGGTGCCGATATGTATACCGCTAAGGAACAACGAGCACGAGACCTAGCTGATGAACGCTCTAACGAGATTATCCGTAAGCTGACCCCTGAGCAACGTCGAGAAGCTCTCAACAACGGGACCCTTCTGTATCAGGATGACCCATACGCTATGGAAGCACTCCGAGTCAAGACTGGTCGTAACGCTGCGTATCTTGTGGACGATGACGTTATGCAGAGGATAAAAGAGGGTGTCTTCCGTACTCGCGAAGAGATGGAAGAGTATCGCCATAGTCGCCTTCAAGAGGGCGCTAAGGTATACGc                                                                                                                                                                                                                      <  1:61829/301‑1 (MQ=255)`  
                                                                                  `aaagagacaggatGTATACCGCTAAGGAACAACGAGCACGAGACCTAGCTGATGAACGCTCTAACGAGATTATCCGTAAGCTGACCCCTGAGCAACGTCGAGAAGCTCTCAACAACGGGACCATTCTGTATCAGGATGACCCATACGCTATGGAAGCACTCCGAGTCAAGACTGGTCGTAACGCTGCGTATCTTGTGGACGATGACGTTATGCAGAGGATAAAAGAGGGTGTCTTCCGTACTCGCGAAGAGATGGAAGAGTATCGCCATAGTCGCCTTCAAGAGGGCGCTAAGGTATACGc                                                                                                                                                                                                                      <  1:161890/290‑1 (MQ=255)`  
                                                                                  `aaagaacaagtatGGAGACCGCCAAGGAAGAACAAGCAAGAGACCTAAGTGAAGAACGCGCTAACGAGATTATCAATAAGCAGACACCTGAGCAACGTCGAGAAGCTTTCAACAACGGGACCCGTCTGAATAAGGATTACCCATACGCTATGGAAGCACACCGAGTCAAGACTGGTCGTAAAGCTGCGTATCTTGTGGACAATGACGTTATGAAGAGGATAAAAGAGGGTGTCTTCCGTACTCGCGAAGAGATGGAAGAGTATCGCCATAGTCGCCTTCAAGAGGGCGCTAAGGTATACGc                                                                                                                                                                                                                      <  1:262953/291‑1 (MQ=255)`  
                                                                                    `ggTGCCGATATGTATACCGCTAAGGAACAACGAGCACGAGACCTAGCTGATGAACGCTCTAACGAGATTATCCGTAAGCTGACCCCTGAGCAACGTCGAGAAGCTCTCAACAACGGGACCCTTCTGTATCAGGATGACCCATACGCTATGGAAGCACTCCGAGTCAAGACTGGTCGTAACGCTGCGTATCTTGTGGACGATGACGTTATGCAGAGGATAAAAGAGGGTGTCTTACGTACTCGCGAAGAGATGGAAGAGTATCGCCATAGTCGCCTTCAAGAGGGGGCTAAGGTATACGCTg                                                                                                                                                                                                                    >  1:308944/1‑301 (MQ=255)`  
                                                                                      `agatgtgtataagagacagaaGGAACAACGAGCACGAGACCTAGCTGATGAACGCTCTAACGAGATTATCCGTAAGCTGACCCCTGAGCAACGTCGAGAAGCTCTCAACAACGGGACCCTTCTGTATCAGGATGACCCATACGCTATGGAAGCACTCCGAGTCAAGACTGGTCGTAACGCTGCGTATCTTGTGGACGATGACGTTATGCAGAGGATAAAAGAGGGTGTCTTCCGTACTCGCGAAGAGATGGAAGAGTATCGCCATAGTCGCCTTCAAGAGGGCGCTAAGGTATACACTGAg                                                                                                                                                                                                                  <  1:48541/282‑1 (MQ=255)`  
                                                                                       `gCCGATATGTATACCGCTAAGGAACAACGAGAACGAGACCTAGCTGATGAACGATCTAACGAGATTATCCGTAAGCTGACCCCTGAGCAACGTCGAGAAGCTCTCAACAACGGGACCCTTCTGTATCAGGATGACCCATACGCTATGGAAGCACTCCGAGTCAAGACTGGTCGTAACGCTGCGTATCTTGTGGACGATGACGTTATGCAGAGGATAAAAGAGGGTGTCTTCCGTACTCGCGAAGAGATGGAAGAGTATCGCCATAGTCGCCTTCAAGAGGGCGCTAAGGTATACGCTGAGc                                                                                                                                                                                                                 <  1:174100/301‑1 (MQ=255)`  
                                                                                           `atatGTATACCGCTAAGGAACAACAAGCACGAGACCTAGCTGATGAACGCTCTAACGAGATTATCCGTAAGCTGACCCCTGAGCAACGTCGAGAAGCTCTCAACAACGGGACCCTTCTGTATCAGGATGACCCATACGCTATGGAAGCACTCCGAGTCAAGACTGGTCGTAACGCTGCGTATCTTGTGGACGATGACGTTATGCAGAGGATAAAGAAGGGTTTCTTCCGTACTCGCGAAGAGATGTAAGAGTATCGCCATAGTCGCCTTCCAGACGGCGCTATGGTATACGCTGAGCAGtt                                                                                                                                                                                                             >  1:167895/1‑301 (MQ=255)`  
                                                                                            `tatGTATACTGCTAAGGAACAACGAGCACGAGACCTAGCTGATGAACGCTCTAACGAGATTATCCGTAAGCTGACCCCTGAGCAACGTCGAGAAGCTCTCAACAACGGGACCCTTCTGTATCAGGATGACCCATACGCTATGGAAGCACTCCGAGTCAAGACTGGTCGTAACGCTGCGTATCTTGTGGACGATGACTTTATGCAGAGGATAAAAGAGGGTGTCTTCCGTACGCTCGAAGAGATGGAAGAGTATCGCCATAGTCGCCTTCAAGAGGGCGCTAAGGTATACGCTGAGCAGttg                                                                                                                                                                                                            >  1:295468/1‑300 (MQ=255)`  
                                                                                            `tatGTATACCGCTAAGGAACAACGAGCACGAGACCTAGCTGATGAACGCTCTAACTAGATTATCCGTAAGCTGACCCCTGAGCAACGTCGAGAAGCTCTCAACAACGGGACCCTTCTGTATCAGGATGACCCATACGCTATGGAAGCACTCCGAGTCAAGACTGGTCGTAACGCTGCGTATCTTGTGGACGATGACGTTATGCAGAGGATAAAATAGGGTGTCTTCCGTACTCGCGAAGAGATGGAAGAGTATCGCCATAGTCGCCTTCAAGAGGGAGATAAGGTATACGCTGAGCAGTTc                                                                                                                                                                                                            >  1:18289/1‑301 (MQ=255)`  
                                                                                               `gTATACCGCTAAGGAACAACGAGCACGAGACCTAGCTGATGAACGCTCTAACGAGATTATCCGTAAGCTGACCCCTGAGCAACGTCGAGAAGCTCTCAACAACGGGACCCTTCTGTATCAGGATGACCCATACGCTATGGAAGCACTCCGAGTCAAGACTGGTCGTAACGCTGCGTATCTTGTGGACGATGATGTTATGCAGAGGATAAAAGAGGGTGTCTTCCGTACTCGCGAAGAGATGGAAGAGTATCGCCATAGTCGCCTTCAAGAGGGCGCTAAGGTATACGcctgtctcttatac                                                                                                                                                                                                         >  1:279954/1‑288 (MQ=255)`  
                                                                                               `gTATACCGCTAAGGAACAACGAGCACGAGACCTAGCTGATGAACGCTCTAACGAGATTATCCGTAAGCTGACCCCTGAGCAACGTCGAGAAGCTCTCAACAACGGGACCATTCTGTATCAGGACGACCCATACGCTATGGAAGCACTCCGAGTCAAGACTGGTCGTAACGCTGCGTATCTTGTGGACGATGACGTTATGCAGAGGATAAAAGAGGGTGTCTTCCGTACTCGCGAAGAGATGGAAGAGTATCGCCATAGTCGCCTTCAAGAGGGCGCTAAGGTATACGCTGAGCAGTTCGGc                                                                                                                                                                                                         >  1:195452/1‑301 (MQ=255)`  
                                                                                                      `gCTAAGGAACAACGAGCACGAGACCTAGCTGATGAACGCTCTAACGAGATTATCCGTAAGCTGACCCCTGAGCAACGTCGAGAAGCTCTCAACAACGGGACCCTTCTGTATCAGGATGACCCATACGCTATGGAAGCACTCCGAGTCAAGACTGGTCGTAACGCTGCGTATCTGGTGGACGATGACGTTATGCAGAGGATAAAAGAGGGTGTCTTCCGTACTCGCGAAGAGATGGAAGAGTATCGCCATAGTCGCCTTCAAGAGGGCGCTAAGGTATACGCTGAGCAGTTCGGCATCGAcc                                                                                                                                                                                                  <  1:40662/301‑1 (MQ=255)`  
                                                                                                            `gAACCACGAGCACGAGACCTAGCTGATGAACGCTCTAACGAGATTATCCGTAAGCTGACCCCTGAGCAACGTCGAGAAGCTCTCAACAACGGGACCCTTCTGTATCAGGATGACACATACGCTATGGAAGCACTCCGAGTCAAGACTGGTCGTAACGCTGCGTATCTTGTGGACGATGACGTTATGCAGAGGATAAAAGAGGGTGTCTTCCGTACTCGCGAAGAGATGGAAGAGTATCGCCATAGTCGCCTTCAAGAGGGCGCTAAGGTATACGCTGAGCAGTTCGGTATCGACCCTGAgg                                                                                                                                                                                            <  1:235421/301‑1 (MQ=255)`  
                                                                                                            `gAACAACGAGCACGAGACCTAGCTGATGAACGCTCTAACGAGATTATCCGTAAGCTGACCCCTGAGCAACGTCGAGAAGCTCTCAACAACGGGACCCTTCTGTATCAGGATGACCCATACGCTATGGAAGCACTCCGAGTCAAGACTGGTCGTAACGCTGCGTATCTTGTGGACGATGACGTTATGCAGAGGATAAAAGAGGGTGTCTTCCGTACTCGCGAAGAGATGGAAGAGTATCGCCATAGTCGCCTTCAAGAGGGCGCTAAGGTATACGCTGAGCAGTTCGGCATCGACCCTGAgg                                                                                                                                                                                            <  1:225062/301‑1 (MQ=255)`  
                                                                                                            `gAACAACGAGCACGAGACCTAGCTGATGAACGCTCTAACAGGATTATCCGTAAGCTGACCCATGAGCAACGTCGAGAAGCTCTCAACAACGGGACCCTTCTGTATCAGGATGACCCATACGCTATGGAAGCACTACGAGTCAAGACTGGTCGTAACGCTGCGTAACTTGTGGACGATGACATTATGCAGAGGATAAAAGAGGGTGTCTTCCGTACTCGCGAAGAGATGGAAGAGTATCGCCATAGTCGCCTTCAAGAGGGCGCTAAGGTATACGCTGAGCAGTTCGGCATCGACCCTGAgg                                                                                                                                                                                            <  1:205772/301‑1 (MQ=255)`  
                                                                                                            `aaacaacGAGCACGAGACCTAGCTGATGAACGCTCTAACGAGATTATCCGTAAGCTGACCCCTGAGCAACGTCGAGAAGCTCTCAACAACGGGACACTTCTGTATCAGGATGACCCATACGCTATGGAAGCACTCCGAGTCAAGACTGGTCGTAACGCTGCGTATCTTGTGGACGATGACGTTATGCAGAGGATAAAAGAGGGTGTCTTCCGTACTCGCGAAGAGATGGAAGAGTATCGCCATAGTCGCCTTCAAGAGGGCGCTAAGGTATACGCTGAGCAGTTCGGCATCGACCCTGAgg                                                                                                                                                                                            <  1:332971/300‑1 (MQ=255)`  
                                                                                                                `aacGAGCACGAGACCTAGCTGATGAACGCTCTAACGAGATTATCCGTAAGCTGACCCCTGAGCAACGTCGAGAAGCTCTCAACAACTGGACCCTTCTGTATCAGGATGACCCATACGCTATGGAAGCACTCCGAGTCAAGACTGGTCGTAACGCTGCGTATCTTGTGGACGATGACGTTATGCAGAGGATAAAAGAGGGTGTCTTCCGTACTCGCGAAGAGATGGAAGAGTATCGCCATAGTCGCCTTCAAGAGGGCGCTAAGGTATACGCTGAGCAGTTCGGCATCGACCCTGAGGACGt                                                                                                                                                                                        <  1:268254/301‑1 (MQ=255)`  
                                                                                                                   `gAGCATGAGACCTAGCTGATGAACGCTCTAACGAGATTATCCGTAAGCTGACCCCTGAGCAACGTCGAGAAGCTCTCAACAACGGGACCCTTCTGTATCAGGATGACCCATACGCTATGGAAGCACTCCGAGTCAAGACTGGTCGTAACGCTGCGTATCTTGTGGACGATGACGTTATGCAGAGGATAAAAGAGGGTGTCTTCCGTACTAGCGAAGAGATGGAAGAGTATCGCCATAGTAGCCTTCAAGAGGGCGCTAAGGTATACGCTGAGCAGTTCGGCATCGACCCTGAGGACGTAGa                                                                                                                                                                                     >  1:176902/1‑301 (MQ=255)`  
                                                                                                                     `gCACGATACCTAGCTGATGAACGCTCTAACGAGATTATCCGTAAGCTGACCCCTGAGCAACGTCGAGAAGCTCTCAACAACGGGACCCTTCTGTATCAGGATGACCCATACGCTATGGAAGCACTCCGAGTCAAGACTGGTCGTAACGCTGCGTATCTTGTGGACGATGACATTATGCAGAGGATAAAAGAGGGTGTCTTCCGTACTCGCGAAGAGATGGAAGAGTATCGCCATAGTCGCCTTCAAGAGGGCGCTAAGGTATACGCTGAGCAGTTCGGCATCGACCCTGAGGACGTTGAtt                                                                                                                                                                                   >  1:270375/1‑301 (MQ=255)`  
                                                                                                                        `cGAGACCTAGCGGATGAACGCTCTAACGAGATTATCCGTAAGCTGACCCCTGAGCAACGTCGAGAAGCTCTCAACAACGGGACCCTTCTGTATCAGGATGACCCATACGCTATGGAAGCACTCCGAGTCAAGACTGGTCGTAACGCTGCGTATCTTGTGGACGATGACGTTATGCAGAGGATAAAAGAGGGTGTCTTCCGTACTCGCGAAGAGATGGAAGAGTATCGCCATAGTCGCCTTCAAGAGGGCGCTAAGGTATACGCTGAGCAGTTCGGCATCGACCCTGAGGACGTTGATTATc                                                                                                                                                                                <  1:158089/301‑1 (MQ=255)`  
                                                                                                                          `agaCCTAGCTGATGAGCGCTCTAACGGGATTATCCGTAAGCTGACCCCTGAGCAACGTCGAGAAGCTCTCACCAACGGGACCCTTCTGTAGCAGGATGACCCATACGCTATGGAAGCACTCCGAGTCAAGACGGGTCGTAACGCTGCGTATCTTGTGGACGATGACGTTATGCAGAGGATAAAAGAGGGTGTCTTCCGTACTCGCGAAGAGATGGAAGAGTATCGCCATAGTAGCCTTCAAGAGGGCGCTAAGGTATACGCTGAGCAGTTCGGCATCGACCATGAGGACGTTGATTAGCAg                                                                                                                                                                              <  1:75132/301‑1 (MQ=255)`  
                                                                                                                          `agaCCTAGCTGATGAACGCTCTAACGAGATTATCCGTAAGCTGACCCCTGAGCAACGTCGAGAAGCTCTCAACAACGGGACCCTTCTGTATCAGGATGACCCATACGCTATGGAAGCACTCCGAGTCAAGACTGGTCGTAACGCTGCGTATCTTGTGGACGATGACGTTATGCAGAGGATAAAAGAGGGTGTCTTCCGTACTCGCGAAGAGATGGAAGAGTATCTCCCTAGTCGCCTTCAAGCGGGCGCTAAGGTCTACGCTTAGCAGTTCGGCATCGAACCTGAGGACGTTGATTATccg                                                                                                                                                                              >  1:327939/1‑299 (MQ=255)`  
                                                                                                                          `agaCCTAGCTGATGAACGCTCTAACGAGATTATCCGTAAGCTGACCCCTGAGCAACGTCGAGAAGCTCTCAACAACGGGACCCTTCTGTATCAGGATGACCCATACGCTATGGAAGCACTCCGAGTCAAGACTGGTCGTAACGCTGCGTATCTTGTGGACGATGACGTTATGCAGAGGATAAAAGAGGGTGTCTTCCGTACTCGCGAAGAGATGGAAGAGTATCGCCATAGTCGCCTTCAAGAGGGCGCTAAGGTATACGCTGAGCAGTTCGGCATCGACCCTGAGGACGTTGATTATcct                                                                                                                                                                              >  1:285907/1‑299 (MQ=255)`  
                                                                                                                          `agaCCTAGCTGATGAACGCTCTAACGAGATTATCCGTAAGCTGACCCCTGAGCAACGTCGAGAAGCTCTCAACAACGGGACCCTTCTGTATCAGGATGACCCATACGCTATGGAAGCACTCCGAGTCAAGACTGGTCGTAACGCTGCGTATCTTGTGGACGATGACGTTATGCAGAGGATAAAAGAGGGTGTATTCCGTACTCGCGAAGAGATGGAAGAGTATCGCCATAGTCGCCTTCAAGAGGGCGCTAAGGTATACGCTGAGCAGTTCGGCATCGACCCTGAGGACGTTGATTATccg                                                                                                                                                                              >  1:91574/1‑299 (MQ=255)`  
                                                                                                                            `aCCTAGCTGATGAACGCTCTAACGAGATTATCCGTAAGCTGACCCCTGAGCAACGTCGATAAGCTCTCAACAACGGGACCCTTCTGTATCAGGATGACCCATACGCTATGGAAGCACTCCGAGTCAAGACTGGTCGTAACGCTGCGTATCTTGTGGACGATGACGTTATGCAGAGGATAAAAGAGGGTGTCTTCCGTACTCGCGAAGAGATGGAAGAGTATCGCCATAGTCGCCTTCAAGAGGGCGCTAAGGTATACGCTGAGCAGTTCGGCATCGACCCTGAGGACGTTGATTAACAGCg                                                                                                                                                                            <  1:52472/301‑1 (MQ=255)`  
                                                                                                                                           `gCTCTAACGAGATTATCCGTAAGCTGACCCCTGAGCAACGTCGAGAAGCTCTCAACAACGGGACCCTTCTGTATCAGGATGACCCATACGCTATGGAAGCACTCCGAGTCAAGACTGGTCGTAACGCTGCGTATCTTGTGGACGATGACGTTATGCAGAGGATAAAAGAGGGTGTCTTCCGTACTCGCGAAGAGATGGAAGAGTATCGCCATAGTCGCCTTCAAGAGGGCGCTAAGGTATATGCTGAGCAGTTCGGCATCGACCCTGAGGACGTTGATTATCAGCGTGGTTTCAACGGGGa                                                                                                                                                             <  1:165674/301‑1 (MQ=255)`  
                                                                                                                                            `ctctAACGAGATTATCCGTAATCTGACCCCTGAGCAGCGTCGAGAAGCTCTCAACAACTGGACCCTTATGTATCAGGATGACCCATACGCTATGGAAGCACTCCGAGTCAAGACTGGTCGTAACGCTGCGTATCTTGTGGACGATGACGTTATGCAGAGGATAAAAGAGGGTTGCTTCCGTACTCGCGAAGAGATGGAAGAGTATCGCCATAGTCGCCTTCAAGAGGGCGCTAAGGTATACGATGAGCAGTTCGGCATCGACCCTGAGGACGTTGATTATCAGCGTGGTTTCAACGGGGAc                                                                                                                                                            <  1:118629/301‑1 (MQ=255)`  
                                                                                                                                            `ctctAACGAGATTATCCGTAAGCTGACCCCTGAGCAACGTCGAGAAGCTCTCAACAACGGGACCCTTCTGTATCAGGATGACCCATACGCTATGGAAGCACTCCGAGTCAAGACTGGTCGTAACGCTGCGTATCTTGTGGACGATGACGTTATGCAGAGGATAAAAGAGGGTGTCTTCCGTACTCGCGAAGAGATGGAAGAGTATCGCCATAGTCGCCTTCAAGAGGGCGCTAAGGTATACGCTGAGCAGTTCGGCATCGACCCTGAGGACGTTGATTATCAGCGTGGTTTCAACGGGGAc                                                                                                                                                            >  1:137453/1‑301 (MQ=255)`  
                                                                                                                                             `tgtAACGAGATTATCCGTAAGCTGACCCCTGAGCAACGTCGAGAAGCTCTCAACAACGGGACCCTTCGGGATCAGGATGACCCATACGCTATGGAAGCACTCCGAGTCAAGACTGGTCGTAACGCTGCGTATCTTGTGGACGATGACGTTATGCAGATGATAAAAGAGGGTGTCTTCCGTACTCGCGAAGAGATGGAAGAGTATCGCCATAGTCGCCTTCAAGAGGGCGCTAAGGTATACGCTGAGCAGTTCGGCATCGACCCTTAGGACGTTGATTATCAGCGTGGTTTCAACGGGGACa                                                                                                                                                           <  1:193321/299‑1 (MQ=255)`  
                                                                                                                                                 `aCGAGATTATCCGTAAGCTGACCCCTGAGCAACGTCGAGAAGCTCTCAACAACGGGACCCTTCTGTATCAGGATGACCCATACGCTATGGAAGCACTCCGAGTCAAGACTGGTCGTAACGCTGCGTATCTTGTGGACGATGACGTTATGCAGAGGATAAAAGAGGGTGTCTTCCGTACTCGCGAAGAGATGGAAGAGTATCGCCATAGTCGCCTTCAAGAGGGCGCTAAGGTATACGCTGAGCAGTTCGGCATCGACCCTGAGGACGTTGATTATCAGCGTGGTTTCAACGGGGACATTAc                                                                                                                                                       <  1:230936/301‑1 (MQ=255)`  
                                                                                                                                                  `cGAGATTATCCGTAAGCTGACCCCTTAGCAACGTCGAGAAGCTCTCAACAACGGGACCCTTCTGTATCAGGATGACCCATACGCTATGGAAGCACTCCGAGTCAAGACTGGTCGTAACGCTGCGTATCTTGTGGACGATGACGTTATGCAGAGGATAAAAGAGGGTGTCTTCCGTACTCGCGAAGAGATGGAAGAGTATCGCCATAGTCGCCTTCAAGAGGGCGCTAAGGTATACGCTGAGCAGTTCGGCATCGACCCTGAGGACGTTGATTATCAGCGTGGTTTCAACGGGGACATTAcc                                                                                                                                                      >  1:249524/1‑301 (MQ=255)`  
                                                                                                                                                    `agaTTATCCGTAAGCTGACCCCTGAGCAACGTCGAGAAGCTCTCAACAACGGGACCCTTCTGTATCAGGATGACCCATACGCTATGGAAGCACTCCGAGTCAAGACTGGTCGTAACGCTGCGTATCTTGTGGACGATGACGTTATGCAGAGGATAAAAGAGGGTGTCTTCCGTACTCGCGAAGAGATGGAAGAGTATCGCCATAGTCGCCTTCAAGAGGGCGCTAAGGTATACGCTGAGCAGTTCGGCATCGACCCTGAGGACGTTGATTATCAGCGTGGTTTCAACGGTGACATTACCGa                                                                                                                                                    >  1:341445/1‑301 (MQ=255)`  
                                                                                                                                                    `agaTTATCCGTAAGCTGACCCCTGAGCAACGTCGAGAAGCTCTCAACAACGGGACCCTTCTGTATCAGGATGACCCATACGCTATGGAAGCACTCCGAGTCAAGACTGGTCGTAACGCTGCGTATCTTGTGGACGATGACGTTATGCAGAGGATAAAAGAGGGTGTCTTCCGTACTCGCGAAGAGATGGAAGAGTATCGCCATAGTCGCCTTCAAGAGGGCGCTAAGGTATACGCTGAGCAGTTCGGCATCGACCCTGAGGACGTTGATTATCAGCGTGGTTTCAACGGGGACATTACCGa                                                                                                                                                    >  1:121825/1‑301 (MQ=255)`  
                                                                                                                                                          `tCCGTAAGCTGACCCCTGAGCAACGTCGAGAAGCTCTCAACAACGGGACCCTTCTGTATCAGGATGACCCATACGCTATGGAAGCACTCCGAGTCAAGACTGGTCGTAACGCTGCGTATCTTGTTGACGATGACGTTATGCAGAGGATAAAAGAGGGTGTCTTCCGTACTCGCGAAGAGATGGAAGAGTATCGCCATAGTCGCCTTCAAGAGGGCGCTAAGGTATACGCTGAGCAGTTCGGCATCGACCCTAAGGACGTTGATTATCAGCGTGGTTTCAACGGGGAcctgtctcttataca                                                                                                                                              >  1:198269/1‑287 (MQ=255)`  
                                                                                                                                                             `gTAAGCTGACCCCTGAGCAACGTCGAGAAGCTCTCAACAACGGGACCCTTCTGTATCAGGATGACCCATACGCTATGGAAGCACTCCGAGTCAAGACTGGTCGTAACGCTGCGTATCTTGTGGACGATGACGTTATGCAGAGGATAAAAGAGGGTGTCTTCCGTACTCGCGAAGAGATGGAAGAGTATCGCCATAGTCGCCTTCAAGAGGGCGCTAAGGTATACGCTGAGCAGTTCGGCATCGACCCTGAGGACGTTGATTATCAGCGTGGTTTCAACGGGGACATTACCGAGCGTAACAt                                                                                                                                           <  1:68044/301‑1 (MQ=255)`  
                                                                                                                                                                 `gCTGACCCCTGAGCAACGTCGAGAAGCTCTCAACAACGGGACCCTTCTGTATCAGGATGACCCATACGCTATGGAAGCACTCCGAGTCAAGACTGGTCGTAACGCTGCGTATCTTGTGGACGATGACGTTATGCAGAGGATAAAAGAGGGTGTCTTCCGTACTCGCGAAGAGATGGAAGAGTATCGCCATAGTCGCCTTCAAGAGGGCGCTAAGGTATACGCTGAGCAGTTCGGCATCGACCCTGAGGACGTTGATTATCAGCGTGGTTTCAACGGGGACATTACCGAGCGTAACATCTCg                                                                                                                                       >  1:113556/1‑301 (MQ=255)`  
                                                                                                                                                                    `ccccccTGAGCAAAGTCGAGAAACTCTCAACCACTGGACCCTTCTGTATCAGGATGACCCGTACGCTATGTAAGCTGTCCGAGTCAAGACTGGTCGTAACGCTGCGTATCTTGTGGACTATGACGTTATGCAGAGGATAAAAGATGGTGTCTTCCGTACTCGCGAAGAGATGGAAGAGTATCGCCATAGTCTCCTTCAAGAGGGCGCTAAGGTATACGCTGAGCAGTTCGGCATCGACCCTGAGTACGTTGATTATCAGCGTGGTTTCAACGGGGACATTACCGAGCGTAACATCTCGCTg                                                                                                                                    <  1:329863/299‑1 (MQ=255)`  
                                                                                                                                                                       `cccTGAGCAACGTCGAGAAGCTCTCAACAACGGGACCCTTCTGTATCAGGATGACCCATACGCTATGGAAGCACTCCGAGTCAAGACTGGTCGTAACGCTGCGTATCTTGTGGACGATGACGTTATGCAGAGGATAAAAGAGGGTGTCTTCCGTACTCGCGAAGAGATGGAAGAGTATCGCCATAGTCGCCTTCAAGAGGGCGCTAAGGTATACGCTGAGCAGTTCGGCATCGACCCTGAGGACGTTGATTATCAGCGTGGTTTCAACGGGGACATTACAGAGCGTAACATCTCGCTGTAt                                                                                                                                 <  1:21245/301‑1 (MQ=255)`  
                                                                                                                                                                         `cggAGCAACGTCGAGAAGCTCGCAACAACGGGACCCTTCTGTATCAGGATGACCCATACGCTATGGAAGCACTCCGAGTCAAGACTGGTCGTAACGCTGCGTATCTTGTGGACGATGACGTTATGCAGAGGATAAAAGAGGGTGTCTTCCGTACTCGCGAAGAGATGGAAGAGTATCGCCATAGTCGCCTTCAAGAGGGCGCTAAGGTATACGCTGAGCAGTTCGGCATCGACCCTTAGGACGTTGATTATCAGCGTGGTTTCAACGGGGACATTACCGAGTGTAACATCTCGCTGTATgg                                                                                                                               <  1:290134/299‑1 (MQ=255)`  
                                                                                                                                                                                     `gagaAGCTCTCAACAACGGGACCCTTCTGTATCAGGATGACCCATACGCTATGGAAGCACTCCGAGTCAAGACTGGTCGTAACGCTGCGTATCTTGTGGACGATGACGTTATGCAGAGGATAAAAGAGGGTGTCTTCCGTACTCGCGAAGAGATGGAAGAGTATCGCCATAGTCGCCTTCAAGAGGGCGCTAAGGTATACGCTGAGCAGTTCGGCATCGACCCTGAGGACGTTGATTATCAGCGTGGTTTCAACGGGGACATTACCGAGCGTAACATCTCGCTGTATGGTGCGCATGATaa                                                                                                                   >  1:221841/1‑301 (MQ=255)`  
                                                                                                                                                                                      `agaAGCTCTCAACAACGGGACCCTTCTGTATCAGGATGACCCATACGCTATGGAAGCACTCCGAGTCAAGACTGGTCGTAACGCTGCGTATCTTGTGGACGATGACGTTATGCAGAGGATAAAAGAGGGTGTCTTCCGTACTCGCGAAGAGATGGAAGAGTATCGCCATAGTCGCCTTCAAGAGGGCGCTAAGGTATACGCTGAGCAGTTCGGCATCGACCCTGAGGACGTTGATTATCAGCGTGGTTTCAACGGGGACATTACCGAGCGTAACATCTCGCTGTATGGTGCGCATGATAAc                                                                                                                  <  1:282321/301‑1 (MQ=255)`  
                                                                                                                                                                                             `ctcAACAACGGGACCCTTCTGTATCAGGATGACCCATACGCTATGGAAGCACTCCGAGTCAAGACTGGTCGTAACGCTGCGTATCTTGTGGACGATGACGTTATGCAGAGGATAAAAGAGGGTGTCTTCCGTACTCGCGAAGAGATGGAAGAGTATCGCCATAGTCGCCTTCAAGAGGGCGCTAAGTTATACGCTGAGCAGTTCGGCATCGACCCTGAGGCCGTTGATTATCAGCGTGGTTTCAACGTGGACATTACCGAGCGTAACATCTCGCTGTATGGTTCTCATGATAACTTCTTgg                                                                                                           >  1:227183/1‑300 (MQ=255)`  
                                                                                                                                                                                                   `aacGGGACCCTTCTGTATCAGGATGACCCATACGCTATGGAAGCACTCCGAGTCAAGACTGGTCGTAACGCTGCGTATCTTGTGGACGATGACGTTATGCAGAGGATAAAAGAGGGTGTCTTCCGTACTCGCGAAGAGATGGAAGAGTATCGCCATAGTCGCCTTCAAGAGGGCGCTAAGGTATACGCTGAGCAGTTCGGCATCGACCCTGAGGACGTTGATTATCAGCGTGGTTTCAACGGGGACATTACCGAGCGTAACATCTCGCTGTATGGTGCGCATGATAAcctgtctcttataa                                                                                                     >  1:56910/1‑288 (MQ=255)`  
                                                                                                                                                                                                       `taaCCCTTCTGTATCAGGATGACCCATACGCTATGGAAGCACTCCGAGTCAAGACTGGTCGTAACGCTGCGTATCTTGTGGACGATGACGTTATGCAGAGGATAAAAGAGGGTGTCTTCCGTACTCGCGAAGAGATGGAAGAGTATCGCCATAGTCGCCTTCAAGAGGGCGCTAAGGTATACGCTGAGCAGTTCTGCATCGACCCTGAGGACGTTGATTATCAGCGTTGTTTCAACGGGGACATTACCGAGCGTAACATCTCGCTGTATGGTGCGCATGATAActtctgtctcttatacac                                                                                                 >  1:77547/3‑288 (MQ=255)`  
                                                                                                                                                                                                         `aCCCTTCTGTATCAGGATGACCCATACGCTATGGAAGCACTCCGAGTCAAGACTGGTCGTAACGCTGCGTATCTTGTGGACGATGACGTTATGCAGAGGATAAAAGAGGGTGTCTTCCGTACTCGCGAAGAGATGGAAGAGTATCGCTATAGTCGCCTTCAAGAGGGCGCTAAGGTATACGCTGAGCAGTTCGGCATCGACCCTGAGGACGTTGATTATCAGCGTGGTTTCAACGGGGACATTACCGAGCGTAAAATCTCGCTGTATGGTGCGCATGATAACTTCTTGAGCCAGCAGGcgc                                                                                               >  1:180344/1‑299 (MQ=255)`  
                                                                                                                                                                                                         `aCCCTTCTGTATCAGGATGACCCATACGCTATGGAAGCACTCCGAGTCAAGACTGGTCGTAACGCTGCGTATCTTGTGGACGATGACGTTATGCAGAGGATAAAAGAGGGTGTCTTCCGTACTCGCGAAGAGATGGAAGAGTATCGCCATAGTCGCCTTCAAGAGGGCGCTAAGGTATACGCTGAGCAGTTCGGCATCGACCCTGAGGACGTTGATTATCAGCGTGGTTTCAACGGGGACATTACCGAGCGTAACATCTCGCTGTATGGTGCGCATGATAACTTCTTGAGCCAGCAAGCTc                                                                                               >  1:190934/1‑301 (MQ=255)`  
                                                                                                                                                                                                                `ggTATCAGGATGACCCATACGCTATGGAAGCACTCCGAGTCAAGACTGGTCGTAACGCTGCGTATCTTGTGTACGATGACGTTATGCAGAGGATAAAAGAGGGTGTCTTCCGTACTCGCGAAGAGATGGAAGAGTATCGCCATAGTCGCCTTCAAGAGGGCGCTAAGGTATACGCTGAGCAGTTCGGCATCGACCCTGAGGACGTTGATTATCAGCGTGGTTTCAACGGGGACATTACCGAGCGTAACATCTCGCTGTATGGTGCGCATGATAACTTCTTGAGCCAGCactgtctcttata                                                                                        >  1:143719/2‑289 (MQ=255)`  
                                                                                                                                                                                                                 `gTATCAGGATGACCCATACGCTATGGAAGCACTCCGAGTCAAGACTGGTCGTAACGCTGCGTATCTTGTGGACGATGACGTTATGCAGAGGATAAAAGAGGGTGTCTTCCGTACTCGCGAAGAGATGGAAGAGTATCGCCATAGTCGCCTTCAAGAGGGCGCTAAGGTATACGTTGAGCAGTTCGGCATCGACCCTGAGGACGTTGATTATCAGCGTGGTTTCAACGGGGACATTACCGAGCGTAACATCTCGCTGTATGGTGCGCATGATAACTTCTTGAGCCAGCAAGCTCAGAAgggg                                                                                       >  1:128451/1‑300 (MQ=255)`  
                                                                                                                                                                                                                 `gTATCAGGATGACCCATACGCTATGGAAGCACTCCGAGTCAAGACTGGTCGTAACGCTGCGTATCTTGTGGACGATGACGTTATGCAGAGGATAAAAGAGGGTGTCTTCCGTACTCGCGAAGAGATGGAAGAGTATCGCCATAGTCGCCTTCAAGAGGGCGCTAAGGTATACGCTGAGCAGTTCGGCATCGACCCTGAGGACGTTGATTATCAGCGTGGTTTCAACGGGGACATTACCGAGCGTAACATCTCGCTGTATGGTGCGCATGATAACTTCTTGAGCCAGCAAGCCCAGAAgggg                                                                                       >  1:256871/1‑300 (MQ=255)`  
                                                                                                                                                                                                                 `gTATCAGGATGACCCATACGCTATGGAAGCACTCCGAGTCAAGACTGGTCGTAACGCTGCGTATCTTGTGGACGATGACGTTATGCAGAGGATAAAAGAGGGTGTCTTCCGTACTCGCGAAGAGATGGAAGAGTATCGCCATAGTCGCCTTCAAGAGGGCGCTAAGGTATACGCTGAGCAGTTCGGCATCGACCCTGAGGACGTTGATTATCAGCGTGGGTTCAACGGGGACATTACCGAGCGTAACATCTCGCTGTATGGTGCGCATGATAAcctgtctcttatacacatctccgagccc                                                                                       >  1:14941/1‑274 (MQ=255)`  
                                                                                                                                                                                                                   `aTCAGGATGACCCATACGCTATGGAAGCACTTCGAGTCAAGACTGGTCGTAACGCTGCGTATCTTGTGGACGATGACGTTATGCAGAGGATAAAAGAGGGTGTCTTCCGTACTCGCGAAGAGATGGAAGAGTATCGCCATAGTCGCCTTCAAGAGGGCGCTAAGGTATACGCTGAGCAGTTCGGCATCGACCCTGAGGACGTTGATTATCAGCGTGGTTCCAACGGGGACATTACCGAGCGTAACATCTAGCTGTATGGTGCGCATGATAACTTCTTGAGCCAGCAAGCTCAGAAGGgcgc                                                                                     >  1:94589/1‑301 (MQ=255)`  
                                                                                                                                                                                                                       `ggATGACCCATACGCTATGGAAGCACTCCGAGTCAAGACTGGTCGTAACGCTGCGTATCTTGTGGACGATGACGTTATGCAGAGGATAAAAGAGGGTGTCTTCCGTACTCGCGAAGAGATGGAAGAGTATCGCCATAGTCGCCTTCAAGAGGGCGCTAAGGTATACGCTGAGCAGTTCGGCATCGACCCTGAGGACGTTGATTATCAGCGTGGTTTCAACGGGGACATTACCGAGCGTAACATCTCGCTGTATGGTGCGCATGATAACTTCTTGAGCCAGCAAGCTCAGAAGGGAGCTATc                                                                                 >  1:124944/1‑301 (MQ=255)`  
                                                                                                                                                                                                                             `cccATACGCTATGGAAGCACTCCGAGTCAAGACTGGTCGTAACGCTGCGTATCTTGTGGACGATGACGTTATGCAGAGGATAAAAGAGGGTGTCTTCCGTACTCGCGAAGAGATGGAAGAGTATCGCCATAGTCGCCTTCAAGAGGGCGCTAAGGTATACGCTGAGCAGTTCGGCATCGACCCTTAGGACGTTGATTATCAGCGTGGTTTCAACGGGGACATTACCGAGCGTCACATCTCGCTGTATGGTGCGCATGATAACTTCTTTAGCCAGCAAGCCCAGAAGGTCGCTATCATGAAc                                                                           >  1:324862/1‑301 (MQ=255)`  
                                                                                                                                                                                                                             `cccATACGCTATGGAAGCACTCCGAGTCAAGACTGGTCGTAACGCTGCGTATCTTGTGGACGATGACGTTATGCAGAGGATAAAAGAGGGTGTCTTCCGTACTCGCGAAGAGATGGAAGAGTATCGCCATAGTAGCCTTCAAGAGGGCGCTAAGGTATACGCTGAGCAGTTCGGCATCGACCCTGAGGACGTTGATTATCAGCGTGGTTTCAACGGGGACATTACCGAGCGTAACATCTCGCTGTATGTTGCGCATGATAACTTCTTGAGCCAGCAAGCTCAGAAGGGCGCTATCATGaaa                                                                           >  1:81693/1‑300 (MQ=255)`  
                                                                                                                                                                                                                             `cccATACGCTATGGAAGCACTCCGAGTCAAGACTGGTCGTAACGCTGCGTATATTGTGGACGATGACGTTATGCAGAGGATAAAAGAGTGTGTCTTCCGTACTCGCGAAGAGATGGAAGAGTATCGCCATAGTCGCCTTCAAGAGGGCGCTAAGGTATACGCTGAGCAGTTCGGCATCGACCCTGAGGACGTTGATTATCAGCGTGGTTTCAACGGGGACATTACCGAGAGTAACATCTCGCTGTATGGTGCGCATGATAACTTCTTGAGCCAGAAAGATAAGAAGGGAGCTATCATGAAc                                                                           >  1:54811/1‑301 (MQ=255)`  
                                                                                                                                                                                                                              `ccATACGCTATGGAAGCACTCCGAGTCAAGACTGGTCGTAACGCTGCGTATCTTGTGGACGATGACGTTATGCAGAGGATAAAAGAGGGTGTCTTCCGTACTCGCGAAGAGATGGAAGAGTATCGCCATAGTCGCCTTCAAGAGGGCGCTAAGGTATACGCTGAGCAGTTCGTCATCGACCCTGAGGACGTTGATTATCAGCGTGGTTTCAACGGGGACATTACCGTTCGTAACATCTCGCTGTATGGTTCGCATGATAACTTCTTGAGCCAGCAAGCTCAGAAGGGCGCTATCATGTAct                                                                          >  1:272735/1‑300 (MQ=255)`  
                                                                                                                                                                                                                                    `gcaaTGGAAGCACTCCTGGTCAAGACGGGTCGTAACGCTGCGTATCTTGTGGACGATGACGTTATGCAGAGGATAAAAGAGGGTGTCTTCCGTACTCGCGAAGAGATGGAAGAGTATCGCCATAGTCGCCTTCAAGAGGGCGCTAAGGTATACGCTGAGCAGTTCGGCATCGACCCTGAGGACGTTGATTATCAGCGTGGTTTCAACGGGGACATTACCTAGCGTAACATCTCGCTGTATGGTGCGCATGATAACTTCTTGAGCCAGCAAGCTCAGAAGGGCGCTATCATGAACAGCCGAg                                                                    <  1:202486/298‑1 (MQ=255)`  
                                                                                                                                                                                                                                     `cTATGGAAGCACTCCGAGTCAAGACTGGTCGTAACGCTGCGTATCTTGTGGACGATGACGTTATGCAGAGGATAAAAGAGGGTGTCTTCCGTACTCGCGAAGAGATGGAAGAGTATCGCCATAGTCGCCTTCAAGAGGGCGCTAAGGTATACGCTGAGCAGTTCGGCATCGACCCTGAGGACGTTGATTATCAGCGTGGTTTCAACGGGGACATTACCGAGCGTAACATCTCGCTGTATGGTGCGCATGATAACTTCTTGAGCCAGCAAGCTCAGAAGGGCGCTATCATGAACAGCCGAgg                                                                   >  1:65388/1‑300 (MQ=255)`  
                                                                                                                                                                                                                                          `gAAGCACTCCGAGTCAAGACTGGTCGTAACGCTGCGTATCTTGTGGACGATGACGTTATGCAGAGGATAAAAGAGGGTGTCTTCCGTACTCGCGAAGAGATGGAAGAGTATCGCCATAGTCGCCTTCAAGAGGGCGCTAAGGTATACGCTGAGCAGTTCGGCATCGACCCTGAGGACGTTGATTATCAGCGTGGTTTCAACGGGGACATTACCGAGCGTAACATCTCGCTGTATGGTGCGCATGATAACTTCTTGAGCCAGCAAGCTCAGAAGGGCGCTATCATGAACAGCCGAGTGGAAc                                                              <  1:215545/301‑1 (MQ=255)`  
                                                                                                                                                                                                                                              `cACTCCGAGTCAAGACTGGTCGTAACGCTGCGTATCTTGTGGACGATGACGTTATGCAGAGGATAAAAGAGGGTGTCTTCCGTACTCGCGAAGAGATGGAAGAGTATCGCCATAGTCGCCTTCAAGAGGGCGCTAAGGTATACGCTGAGCAGTTCGGCATCGACCCTGAGGACGTTTATTATCAGCGTGGTTTCAACGGGGACATTACCGAGCGTAACATCTCGCTGTATGGTGCGCATGATAACTTCTTGAGCCAGCAAGCTCAGAAGGGCGCTATCATGAACAGCCGAGTGGCACTCaa                                                          >  1:313899/1‑301 (MQ=255)`  
                                                                                                                                                                                                                                               `aCTCCGAGTCAAGACTGGTCGTAACGCTGCGTATCTTGTGGACGATGACGTTATGCAGAGGATAAAAGAGGGTGTCTTCCGTACTCGCGAAAAGATGGAAGAGTATCGCCATAGTCGCCTTCAAGAGGGCGCTAAGGTATACGCTGAGCAGTTCGGCATCGACCCTGAGGACGTTGATTATCAGCGTGGTTTCAACGGGGACATTACCGAGCGTAACATCTCGCTGTATGGTGCGCATGATAACTTCTTGAGCAAGCAAGCTCAGAAGGGCGCTATCATGAACAGCCGAGTGGAACTCAAc                                                         <  1:287842/301‑1 (MQ=255)`  
                                                                                                                                                                                                                                                `cTCCTAGTCAAGACTGGTCGTAACGCTGCGTATCTTGTGGACGATGACGTTATGCAGAGGATAAAAGAGGGTGTCTTCCGTACTCGCGAAGAGATGGAAGAGTATCGCCATAGTCGCCTTCAAGAGGTCGCTAAGGTATACGCTGAGCAGTTCGGCATCGACCCTGAGGACGTTGATTATCAGCGTGGTTTCAACGGGGACATTACCGAGCGTAACATCTCGCTGTATGGTGCGCATGATAACTTCTTGAGCCAGCCAGCCCAGAAGGGCGCTATCATGAACAGCCGAGTGGAACTCAACg                                                        >  1:321765/1‑301 (MQ=255)`  
                                                                                                                                                                                                                                                         `ctaggagatgtgtataagagacagcTTGTGGACGATGACGTTATGCAGAGGAAAAAAGAGGGTGTCTTCCGTACTCGCGAAGAGATGGAAGAGTATCGCCATAGTCGCCTTCAAGAGGGCGCTAAGGTATACGCTGAGCAGTTCGGCATCGACCCTGAGGACGTTGATTATCAGCGTGGTTTCAACGGGGACATTACCGAGCGTAACATCTCGCTGTATGGTGCGCATGATAACTTCTTGAGCCAGCAAGCTCAGAAGGGCGCTATCATGAACAGCCGAGTGGAACTCAACGGTGTCCTTc                                               <  1:338740/277‑1 (MQ=255)`  
                                                                                                                                                                                                                                                         `aaGACTGGTCGTAACGCTGCGTATCTTGTGGACGATGACGTTATGCAGAGGATAAAAGAGGGTGTCTTCCGTACTCGCTAAGAGATGGAAGAGTATCGCCATAGTCGCCTTCAAGAGGGCGCTAAGGTATACGCTGAGCAGTTCGGCATCGACCCTGAGGACGTTGATTATCAGCGTGGTTTCAACGGGGACATTACCGAGCGTAACATCTCGCTGTATGGTGCGCATGATAACTTCTTGAGCCAGCAAGCTCAGAAGGGCGCTATCATGAACAGCCGAGTGGAACTCAACGGTGTCCTTc                                               <  1:265295/301‑1 (MQ=255)`  
                                                                                                                                                                                                                                                          `aGACTGGTCGTAACGCTGCGTATCTTGTGGACGATGACGTTATGCAGAGGATAAAAGAGGGTGTCTTCCGTACTCGCGAAGAGATGGAAGAGTATCGCCATAGTCGCCTTCAAGAGGGCGCTAAGGTATACGCTGAGCAGTTCGGCATCGACCCTGAGGACGTTGATTATCAGCGTGGTTTCAACGGGGACATTACCGAGCGTAACAGCTCGCTGTATGGTGCGCATGATAACTTCTTGAGCCAGCAAGCTCAGAAGGGCGCTATCATGAACAGCCGAGTGGAACTCAACGGTGTCCTTCa                                              >  1:186470/1‑301 (MQ=255)`  
                                                                                                                                                                                                                                                                `gTCGTAACGCTGCGTATCTTGTGGACGATGACGTTATGCAGATGATAAAAGAGGGTGTCTTCCGTACTCGCGAAGAGATGGAAGAGTATCGCCATAGTCGCCTTCAAGAGGGCGCTAAGGTATACGCTGAGCAGTTCGTCATCGACCCTGAGGACGTTGATTATCAGCGTGGTTTCAACGGGGACATTACCGAGCGTAACATCTCGCTGTATGGTGCGCATGATAACTTCTTGAGCCAGCAAGCTCAGAATGGCGCTATCATGAACAGCCGAGTGGAACTCAACGGTGTCCTTCAAGAacc                                        >  1:152591/1‑301 (MQ=255)`  
                                                                                                                                                                                                                                                                        `gCTGCGTATCTTGTGGACGATGACGTTATGCAGAGGATAAAAGAGGGTGTCGTCCGTACTCGCGAAGAGATGGAAGAGTATCGCCATAGTCGCCTTCAAGAGGGCGCTAAGGTATACGCTGAGCAGTTCGGCATCGACCCTGAGGACGTTGATTATCAGCGTGGTTTCAACGGGGACATTACCGAGCGTAACATCTCGCTGTATGGTGCGCATGATAACTTCTTGAGCCAGCAAGCTAAGAAGGGCGCTATCATGAACAGCCGAGTGGAACTCAACGGTGTCCTTCAAGACCCTGATAtgc                                <  1:11586/301‑1 (MQ=255)`  
                                                                                                                                                                                                                                                                          `tGCGTATCTTGTGGACGATGACGTTATGCAGAGGATAAAAGAGGGTGTCTTCCGTACTCGCGAAGAGATGGAAGAGTATCGCCATAGTCGCCTTCAAGAGGGCGCTAAGGTATACGCTGAGCAGTTCAGCATCGACCCTGAGGACGTTGATTATCAGCGTGGTTTCAACGGGGACATTACCGAGCGTAACATCTCGCTGTATGGTGCGCATGATAACTTCTTGAGCCAGCAAGCTCAGAAGGGCGCTATCATGAACAGCCGAGTGGAACTCAACGGTGTCCTTCAAGACCCTGATAtgctg                              <  1:85827/301‑1 (MQ=255)`  
                                                                                                                                                                                                                                                                                 `cTTGTGGACGATGACGTTATGCAGAGGATAAAAGAGGGTGTCTTCCGTACTCGCGAAGAGATGGAAGAGTATCGCCATAGTCGCCTTCAAGAGGGCGCTAAGGTATACGCTGAGCAGTTCGGCATCGACCCTGAGGACGTTGATTATCAGCGTTGTTTCAACGGGGACATTACCGAGCGTAACATCTCGCTGTATGGTGCGCATGATAACTTCTTGAGCCAGCAAGCTCAGAAGGGCGCTATCATGAACAGCCGAGTGGAAATCAACGGTGTCCTTCAAGACCCTGATAtgcctgtctctt                       >  1:134557/1‑292 (MQ=255)`  
                                                                                                                                                                                                                                                                                  `ttGTGGACGATGACGTTATGCAGAGGATAAAAGAGGGTGTCTTCCGTACTCTCGAAGAGCTGTAAGAGTATCGCCATAGTCTCCTTCAAGAGGGCTCTAAGGTATACGCTTAGCAGTTCGGCATCGACCCTGAGGACGTTGATTATCAGCGTGGTTTCAACGGGGACATTACCGAGCGTAACATCTCGCTGTATGGTGCGCATGATAACTTCTTGAGCCAGCAAGCTCAGAAGGGCGCTATCATGAACAGCCGAGTGGAACTCAACGGTGTCCTTCAAGACCCTGATATGCTGCGTCGTcc                      <  1:293480/301‑1 (MQ=255)`  
                                                                                                                                                                                                                                                                                  `ttGTGGACGATGACGTTATGCAGAGGATAAAAGAGGGTGTCTTCCGTACTCGCGAAGAGATGGAAGAGTATCGCCATAGTCGCCTTCAAGAGGGCGCTAAGGTATACGCTGAGCAGTTCGGCATCGACCCTGAGGACGTTGATTATCAGCGTGGTTTCAACGGGGACATTACCGAGCGTAACATCTCGCTGTATGGTGCGCATGATAACTTCTTGAGCCAGCAAGCTAAGAAGGGCGCTATCATGAACAGCCGAGTGGAACTCAACGGTGTCCTTCAAGACCCTGATATGCTGCGTCGTcc                      <  1:260493/301‑1 (MQ=255)`  
                                                                                                                                                                                                                                                                                   `tgtgGACGATGACGTTATGCAGAGGATAAAAGAGGGTGTCTTCCGTACTCGCGAAGAGATGGAAGAGTATCGCCATAGTCGCCTTCAAGAGGGCGCTAAGGTATACGCTGAGCAGTTCGGCATCGACCCTGAGGACGTTGATTATCAGCGTGGTTTCAACGGGGACATTACCGAGCGTAACATCTCGCTGTATGGTGCGCATGATAACTTCTTGAGCCAGCAAGCTCAGAAGGGCGCTATCATGAACAGCCGAGTGGAACTCAACGGTGTCTGTCAAGaactgtctcttatacacatctcc                     >  1:316438/1‑279 (MQ=255)`  
                                                                                                                                                                                                                                                                                       `gACGATGACGTTATGCAGAGGATAAAAGAGGGTGTCTTCCGTACTCGCGAAGAGATGGAAGAGTATCGCCATAGTCGCCTTCAAGAGGGCGCTAAGGTATACGCTGAGCAGTTCGGCATCGACCCTGAGGACGTTGATTATCAGCGTGGTTTCAACGGGGACATTACCGAGCGTAACATCTCGCTGTATGGTTCGCATGATAACTTCTTGAGCCAGCAAGCTCAGAAGGGCGCTATCATGAACAGCCGAGTGGAACTCAACGGTGTCCTTCAAGACCCTGATAtgcctgtctcttatacac                 >  1:35136/1‑286 (MQ=255)`  
                                                                                                                                                                                                                                                                                               `cGTTATGCAGAGGATAAAAGAGGGTGTCTTCCGTACTCGCGCAGAGATGGAAGAGTATCGCCATATTCTCCTTCCAGAGGGCGCTCAGGTATACGCTGAGCAGTTCGGCATCGACCCTTAGGACGTTGATTATCAGCGTGGTTTCAACGGGGACATTACCGAGCGTAACATCTCGCTGTATGGTGCTCATGATAACTTCTTGAGCCATCAAGCTCAGAAGTGCGCTATCATGAACAGCCGATTGGAACTCAACGGTGTCCTTCAAGACCCTGATATTCTGCGTCGTCCAGACTCTGCCGAc         >  1:155340/1‑301 (MQ=255)`  
                                                                                                                                                                                                                                                                                                `tataagagacagaTAAAAGAGGGTGTCTTCCGTACTCGCGAAGAGATGGAAGAGTATCGCCATAGTCGCCGTCAAGAGGGCGCTAAGGTATACGCTGAGCAGTTCGGCATCGACCCTGAGGACGTTGATTATCAGCGTGGTTTCAACGGGGACATTACCGAGCGTAACATCTCGCTGTATGGTGCGCATGATAACTTCTTGAGCCAGCAAGCTCAGAAGGGCGCTATCATGAACAGCCGAGTGGAACTCAACGGTGTCCTTCAAGACCCTGATATGCTGCGTCGTCCAGACTCTGCTGAct        <  1:88819/291‑1 (MQ=255)`  
                                                                                                                                                                                                                                                                                                `gTTATGCAGAGGATAAAAGAGGGTGTCTTCCGTACTCGCGAAGAGATGGAAGAGTATCGCCATAGTCGCCTTCAAGAGGGCGCTAAGGTATACGCTGAGCAGTTCGGCATCGACCCTGAGGACGTTGATTATCAGCGTGGTTTCAACGGGGACATTACCGAGCGTAACATCTCGCTGTATGGTGCGCATGATAACTTCTTGAGCCAGCAAGCTCAGAAGGGCGCTATCATGAACAGCCGAGTGGAACTCAACGGTGTCCTTCAAGACCCTGATATGCTGCGTCGTCCAGACTCTGCTGAct        >  1:140060/1‑301 (MQ=255)`  
                                                                                                                                                                                                                                                                                                `gTTATGCAGAGGATAAAAGAGGGTGTCTTCCGTACTCGCGAAGAGATGGAAGAGTATCGCCATAGTCGCCTTCAAGAGGGCGCTAAGGTATACGCTGAGCAGTTCGGCATCGACCCTGAGGACGTTGATTATCAGCGTGGTTTCAACGGGGACATTACCGAGCGTAACATCTCGCTGTATGGTGCGCATGATAACTTCTTGAGCCAGCAAGCTCAGAAGGGCGCTATCATGAACAGCCGAGTGGAACTCAACGGTGTCCTTCAAGACCCTGATATGCTGCGTCGTCCAGACTCTGCTGAct        >  1:33576/1‑301 (MQ=255)`  
                                                                                                                                                                                                                                                                                                `gTTATGCAGAGGATAAAAGAGGGTGTCTTCCGTACTCGCGAAGAGATGGAAGAGTATCGCCATAGTCGCCTTCAAGAGGGCGCTAAGGTATACGCTGAGCAGTTCGGCATCGACCCTGAGGACGTTGATTATCAGCGTGGTTTCAACGGGGACATTACCGAGCGTAACATCTCGCTGTATGGTGCGCATGATAACTTCTTGAGCCAGCAAGCTCAGAAGGGCGCTATCATGAACAGCCGAGTGGAACTCAACGGTGTCCTTCAAGAACCAGATAGCCTGCGTCGTCCAGAATCTGCTGAcg        >  1:146115/1‑300 (MQ=255)`  
                                                                                                                                                                                                                                                                                                   `aTGCATAGGATAAAAGATGGTGTCTTCCGTACTCGCGAAGAGATGGAAGAGTATCTCCATAGTCGCCTTCAAGAGGGCGCTAAGGTATACGCTGAGCAGTTCGGCATCGACCCTGAGGACGTTGATTATCAGCGTGGTTTCAACGGGTACATTACCGAGCGTAACATCTCGCTGTATGGTTCGCATGATAACTTCTTGAGCCAGCAAGCTCAGAAGGGCTCTATCATGAACAGCCGAGTGGAACTCAACGGTGTCCTTCAAGACCCTGATATTCTGCGTCGTCCAGCATCTGCTTGcttct     >  1:209499/1‑301 (MQ=255)`  

                                                                                                                                                                                                                                                                                                          `|`                                                                                                                                                                                                                                                                                                          
`GGTATGGGCTATCGTGCAGCAACCACTCAGGCCGAACAGCCAAGGTCAAGCCTATTGGACACCATTGGTCGGTTCGCTAAGGCTGGTGCCGATATGTATACCGCTAAGGAACAACGAGCACGAGACCTAGCTGATGAACGCTCTAACGAGATTATCCGTAAGCTGACCCCTGAGCAACGTCGAGAAGCTCTCAACAACGGGACCCTTCTGTATCAGGATGACCCATACGCTATGGAAGCACTCCGAGTCAAGACTGGTCGTAACGCTGCGTATCTTGTGGACGATGACGTTATGCAGAAGATAAAAGAGGGTGTCTTCCGTACTCGCGAAGAGATGGAAGAGTATCGCCATAGTCGCCTTCAAGAGGGCGCTAAGGTATACGCTGAGCAGTTCGGCATCGACCCTGAGGACGTTGATTATCAGCGTGGTTTCAACGGGGACATTACCGAGCGTAACATCTCGCTGTATGGTGCGCATGATAACTTCTTGAGCCAGCAAGCTCAGAAGGGCGCTATCATGAACAGCCGAGTGGAACTCAACGGTGTCCTTCAAGACCCTGATATGCTGCGTCGTCCAGACTCTGCTGACTTCTTTG  >  NC_001604.1/28394‑28988`

  

Alignment Legend

Aligned base mismatch/match (shaded by quality score): `ATCG/ATCG` `< 3 ≤` `ATCG/ATCG` `< 10 ≤` `ATCG/ATCG` `< 23 ≤` `ATCG/ATCG` `< 34 ≤` `ATCG/ATCG` `< 38 ≤` `ATCG/ATCG`

Unaligned base: `atcg`    Masked matching base: `atcg`    Alignment gap: `‑`    Deleted base: `‑`
